# Annotations

**Published:** 1909-12-04

**Authors:** 


### ANNOTATIONS.

### The Princess as Patient.

Besides affording material for effective paragraphs in the daily papers, the visit of a Princess to St. George's Hospital in the character of a casualty patient is of interest in other ways, and is, in fact, a sign of the times of no little significance. It illustrates that the voluntary hospitals are a refuge for those who have sustained accident, even though, as in the present instance, of no worse degree than an inconvenient fishbone, from one end of the social scale to the other; that public confidence in the hospitals is so universal that everybody's first instinct when an emergency arises is to seek their aid; and that even members of the Royal Family, which has so often displayed a keen interest in, and rendered personal service to the hospitals, may sometimes receive in person practical proof of the value of these institutions to all classes. Nor can the most puritanical regard this as hospital abuse. The functions of a hospital almoner are always limited to those who come regularly for treatment; casualty patients are exempt, necessarily and rightly, from inquiry into anything except the condition from which they suffer. Moreover, an established custom ordains that anyone suffering from accident who is taken to a hospital afterwards makes an adequate and substantial donation to its funds. Indeed, when the reorganised system of medical relief for all classes, foreshadowed in the report of the Poor Law Commission, comes into being, some system will probably be introduced to meet these cases. The Princess acted, no doubt, on the impulse of the moment; but, none the less, she has unconsciously testified to her own and the public confidence in the efficiency of the voluntary hospitals.

### Juvenile Smoking.

It is not only the House of Commons that will congratulate Mr. Hobhouse on feeling able to say that "the effect of the Act passed last year dealing with juvenile smoking under 16" was a real cause of the diminished consumption of tobacco. This is a capital answer to the amateur "reformers" who are always saying that you cannot make a man sober (or anything else) by Act of Parliament. It is unfortunate that the medical profession has no direct means of putting to the test the alleged good results of the Act in question. While Mr. Hobhouse has the statistics for many years of tobacco consumption before him, with the fact of the Act of 1909 to explain, perhaps, the change for the better in this year's figures, the medical profession has no figures as to the extent and effects on the young of excessive smoking. Every year the Government is extending the responsibilities of medical men by creating fresh exacting posts for them; it would be wise then for the guardians of the nation's health in their turn to become energetic critics and inspectors of the effect of Government measures, and of the truth of Ministerial statements in any way relating to public health. The present is a typical instance of the cases in which medical testimony alone would be of any real value.

### Mr. Burns's Housing Bill.

By the time this number of The Hospital appears Mr. Burns's Housing and Town Planning Bill will probably have received the Royal assent. It is of interest to medical men, if only because by it the importance and the duties of the medical officer of health are still more fully recognised. Very properly the medical officer of health has now become a very important person, and, according to the Bill, every County Council is required to employ his services. The county itself, in matters of public health and sanitation, will become his province, and he has power to requisition all reasonable information from his lieutenants, the district medical officers of health in the county. He will be brought into direct contact with the Local Government Board by the provision which requires him to report what has been done, and what must yet be done, to make the Housing Acts effective. These are the principal provisions of Mr. Burns's Bill which affect the medical profession, and we congratulate Mr. Burns on recognising that one of the best weapons his department possesses in its struggle with the insanitation of urban and rural districts is the medical man in public service.

### Surgeon and Chiropodist.

It is a very curious thing, and in view of present-day political tendencies, perhaps a hopeful one, that in the most democratic politics the status of the medical profession is the highest. In the Scandinavian States, which, though monarchical, are by far the most democratic in Europe, medical men command respect and political influence much greater than in this country, and we fancy the same is the case in our antipodean colonies. Thus there has recently been decided a libel action in Melbourne which might have had a different result had it been tried in this country, because public opinion is more alive in Australasia than in Britain to the public utility of honest and fearless criticism of quacks and adventurers by the medical profession. What happened was this: A philanthropist took an interest in a club-footed boy, and told a doctor he was willing to collect £30 if the latter would undertake the case. This proposal was refused, but instead the boy was taken into hospital and treated free. The boy's mother then very unwisely removed him from hospital because a self-styled "foot specialist"—an ex-bootmaker—promised a cure if the £30 were forthcoming. The same philanthropist once more started to collect this sum, and actually approached the doctor for a subscription. The latter replied denouncing the chiropodist as an impostor, and obtained a verdict when the latter subsequently brought a libel action against him. The judge found that in using this language the defendant was rendering a public service, and it is difficult not to adopt the same view. All the same, the principle of allowing fools to take the consequence of their folly, and of protecting unqualified and dangerous charlatans, is so well established here that it is by no means certain that such a common-sense view would have prevailed had the case occurred in this country.

